# Nutraceuticals and Human Health and Disease

**DOI:** 10.3390/nu17091432

**Published:** 2025-04-24

**Authors:** Francesca Oppedisano

**Affiliations:** Department of Health Sciences, Institute of Research for Food Safety and Health (IRC-FSH), University “Magna Græcia” of Catanzaro, 88100 Catanzaro, Italy; oppedisanof@libero.it

A “possible beneficial role” for health is indicated by the coordinated and integrated activity of biologically active chemicals and nutritional components, as nutraceuticals [1]. Nutraceuticals are derived from either plant or animal sources, and, currently, researchers around the world are endeavoring to completely elucidate their mechanism of action, safety, and effectiveness by supporting their role with clinical data [1].

Nutraceuticals and functional foods can reduce the risk of the onset and worsening of numerous pathologies by acting in parallel with pharmacological therapy, as adjuvants in case of treatment failure, or in situations in which pharmacological therapy cannot be used.

Numerous studies have documented in the literature that natural compounds can affect the expression or activity of proteins and enzymes involved in different cellular and, specifically, mitochondrial functions. This interaction can have beneficial effects on altered metabolic pathways in a variety of pathologies [2,3].

In the Southern Italian region, the Mediterranean diet (Md), which is considered to be a part of the “intangible cultural heritage of humanity”, is linked to better health, particularly in terms of lower rates of diabetes mellitus, cancer, and—most significantly—coronary heart disease [4]. Specifically, Md has a positive impact on the development and progression of heart disease due to the decreased consumption of red meat and increased consumption of plant-based foods and olive oil [4]. For example, bergamot (*Citrus bergamia Risso et Poiteau*), thanks to its high concentration of polyphenols, is used in nutraceutical integration in numerous pathologies. BPF, obtained from the juice and albedo of bergamot, is a fraction rich in polyphenols known for its protective effects on human health, demonstrated through its antioxidant, anti-inflammatory, hypolipidemic, and hypoglycemic effects [4]. In fact, BPF seems to have a beneficial role in the pathophysiological mechanisms of diabetic cardiomyopathy, particularly in mitochondrial and sarcoplasmic dysfunctions [4]. BPF’s positive effects on oxidative metabolism and mitochondrial bioenergetics have recently been documented, adding to the list of biological targets for its favorable impacts [4]. In addition, BPF could be incorporated into pharmacological therapy for cancer and schizophrenia [4,5].

This Special Issue, “Nutraceuticals and Human Health and Disease”, is devoted to the function and future directions of nutraceuticals in human health, analyzed from a variety of perspectives, from efficacy studies to clinical trials, in terms of their analytical aspects and their positive effects on health issues.

To demonstrate the efficacy of nutraceuticals in human health, Rauch et al. (Contributor 1) studied and proposed the intragastric gavage of exogenous ketones as KEMCT (a mix of 1,3-butanediol-acetoacetate diester/ketone ester/KE and medium-chain triglyceride/MCT oil at a 1:1 ratio) to alleviate anxiety disorder symptoms. Min et al. (Contributor 2) evaluated the anti-Human coronavirus OC43 (HCoV-OC43) effects of auraptene (7-geranyloxycoumarin), a prenyloxycoumarin abundantly present in *Citrus* spp. fruits, demonstrating its efficacy as a therapeutic agent against human coronavirus.

Additionally, Pouchieu et al. (Contributor 3) evaluated the acute effects of a saffron extract (Safr’InsideTM) and its primary volatile compound, safranal, on biological and psychological stress responses in a randomized, double-blind, placebo-controlled cross-over study involving 19 healthy volunteers who underwent a laboratory stress procedure.

The effects of citrate on epithelial cells with intact cystic fibrosis (CF) transmembrane conductance regulator (CFTR) and defective ΔF508-CFTR were assessed by Borkenhagen and Prehm (Contributor 4). These studies showed that citrate is an activator of ΔF508-CFTR and increases export by defective ΔF508-CFTR into the extracellular matrix of epithelial cells.

The antiproliferative effect of fruits and vegetables on cancer, and, in particular, on breast cancer, has also been demonstrated by the studies of Rhman et al. (Contributor 5). In this research, Rhman et al. reported their results obtained on MCF-7 breast cancer cells treated with quercetin (Que) and naringenin (Nar). In particular, they demonstrated the synergistic effects of Que and Nar (CoQN) in inducing oxidative stress and apoptosis in these cells.

Furthermore, the main chemical and biological characteristics of alpha-lipoic acid (ALA) in glucose metabolism have been reported by Capece et al. (Contributor 6). In particular, the antioxidant activity of ALA and its role in the modulation of insulin sensitivity and secretion and in symptomatic peripheral diabetic polyneuropathy are described. In addition, Capece et al. have given a possible explanation for why subjects taking ALA are at risk of developing insulin autoimmune syndrome (IAS).

Therefore, as reported in the review by Puri et al. (Contributors 7), nutraceuticals, in addition to being important from a nutritional point of view, are assuming a central role in the prevention and treatment of various diseases, supporting classic pharmacological therapies, and also reducing radiotherapy- and chemotherapy-related side effects in cancer patients. To enhance these beneficial effects on human health, researchers are using nanotechnologies to improve micronized dietary product and nutraceutical supplement production.

Furthermore, it is known that the intestinal microbiota and its alterations can be involved in numerous diseases, such as metabolic syndrome and acne. Ilari et al. (Contributors 8), in their systematic review, search for microorganisms that may contribute to both metabolic syndrome and acne and report data from the literature relating to the efficacy of polyphenols in metabolic syndrome treatments. In particular, the authors focus their attention on the prebiotic characteristics of polyphenols in promoting intestinal eubiosis in individuals with metabolic syndrome. On the contrary, there are no studies in the literature relating to the specific efficacy of polyphenols for acne treatment.

In light of all this, knowledge of the biochemical relationships between known and unknown natural compounds and cellular metabolism in both health and sickness can aid in the creation of new and innovative treatment strategies.
